# PD-L1^+^MDSCs are increased in HCC patients and induced by soluble factor in the tumor microenvironment

**DOI:** 10.1038/srep39296

**Published:** 2016-12-14

**Authors:** Tomoaki Iwata, Yasuteru Kondo, Osamu Kimura, Tatsuki Morosawa, Yasuyuki Fujisaka, Teruyuki Umetsu, Takayuki Kogure, Jun Inoue, Yu Nakagome, Tooru Shimosegawa

**Affiliations:** 1 Division of Gastroenterology, Tohoku University Hospital, 1-1 Seiryo, Aoba, Sendai City, 980-8574, Miyagi, Japan; 2 Department of Hepatology, Sendai Kousei Hospital, 4-15 Hirose, Aoba, Sendai City, 980-0873, Miyagi, Japan

## Abstract

Myeloid-derived suppressor cells (MDSCs) could have important roles in immune regulation, and MDSCs can be induced in patients with various malignant tumors. The immune-suppressive functions of MDSCs in hepatocellular carcinoma (HCC) patients have not been clarified. Therefore, we tried to analyze the biological significance of MDSCs in HCC patients. We quantified PD-L1^+^MDSCs of HCC patients in various conditions by using multi-color flow cytometry analysis. PBMCs from HCC patients contained significantly higher percentages of PD-L1^+^MDSCs in comparison to those from healthy subjects (*p* < 0.001). The percentages of PD-L1^+^MDSCs were reduced by curative treatment for HCC (*p* < 0.05), and the percentages of PD-L1^+^MDSCs before treatment were inversely correlated with disease-free survival time. After we cocultivated PBMCs and several liver cancer cell lines in a transwell coculture system, the percentages of PD-L1^+^MDSCs were significantly increased compared with control (*p* < 0.05). The expression of M-CSF and VEGFA was higher in the cell lines that strongly induced PD-L1^+^MDSCs. Peripheral blood from HCC patients had significantly higher percentages of PD-L1^+^MDSCs in comparison to those of healthy subjects, and the percentages of PD-L1^+^MDSCs were reduced by HCC treatment, suggesting that we might use PD-L1^+^MDSCs as a new biomarker of HCC.

Hepatocellular carcinoma (HCC) is the fifth leading cause of cancer and second leading cause of cancer-related mortality worldwide^1^,^2^. Approximately 700,000 new cases of HCC are reported, and more than 600,000 deaths are associated with HCC every year worldwide^3^. HCC frequently occurs in patients with cirrhosis from HBV and/or HCV persistent infection^4^.

The efficacy of therapeutic regimens such as surgery, trans arterial chemo-embolization (TAE), radiofrequency ablation (RFA), radiation and sorafenib is limited since immunological abnormalities in the tumor microenvironment are involved in the development and progression of HCC^5^,^6^. Therefore, immunotherapies could become important options for treating HCC. It has been reported that HCC can induce a suppressive network to evade the host immune response^7^,^8^. Several kinds of immune suppressor cells, including regulatory T cells (Tregs), myeloid-derived suppressor cells (MDSCs) and tumor-associated macrophages (TAMs), contribute to the immune suppression in HCC patients^9^. Moreover, we have reported that background liver diseases such as chronic hepatitis B and chronic hepatitis C could suppress immune reactions by various mechanisms^10^,^11^,^12^,^13^,^14^.

MDSCs are a heterogeneous population of myeloid cells and have recently been recognized as a subset of innate immune cells that can alter adaptive immunity and produce immunosuppression^15^. Granulocytic MDSCs and monocytic MDSCs are present in MDSCs^16^. Granulocytic MDSCs are described as CD33^dim^HLA-DR^−^CD66^+^, and monocytic MDSCs are described as CD33^+^HLA-DR^low/−^CD11b^+^CD14^+^ cells in human. MDSCs can suppress immune reactions by various mechanisms. MDSCs inhibit T cell effector functions by the arginase 1 (ARG-1)-mediated depletion of L-arginine^17^, inducible nitric oxide synthase (iNOS) and NADPH oxidase (NOX2) production of reactive nitrogen and oxygen species^18^,^19^, vascular endothelial growth factor (VEGF) over-expression^20^, and the expansion of Tregs populations^21^,^22^. We showed that MDSCs express surface programmed death ligand 1 (PD-L1) molecules in peripheral blood mononuclear cells (PBMCs) and tumor infiltrating lymphocyte (TILs) from HCC patients. It was reported that PD-L1 possessed the dual functions of co-stimulation of naive T cells via an as yet unidentified receptor, and co-inhibition of activated effector T cells through PD-1 receptor^23^. Recent studies showed that MDSCs could contribute to the induction and enhancement of Tregs by the interaction between PD-1 on Tregs and PD-L1 on MDSCs^9^.

HCC is a type of malignant tumor that is easy to recur, requiring repeated treatment. Selection of the appropriate therapy for HCC should take into account the tumor size and location, underlying liver function, the presence or absence of cirrhosis, and the performance status of the patient. Surgical resection, TACE, RFA, radiation, sorafenib, and liver transplantation are standard treatments for HCC. To improve the prognosis of patients with HCC, early detection of HCC is very important^24^. Accordingly, biomarkers in blood for the screening, prediction of prognosis and monitoring of the response to therapy would make an important contribution to the management of HCC patients. Alpha fetoprotein (AFP) is the most widely used and broadly known biomarker for HCC. Similarly, Lens culinaris agglutin-reactive AFP (AFP-L3) and prothrombin induced by vitamin K absence II (PIVKA II) are widely used and broadly known^25^. However, any single biomarker will likely be limited by suboptimal sensitivity. In this study, we report the induction mechanisms of MDSCs in HCC patients. Moreover, we found that the quantification of PD-L1^+^MDSCs could serve as a significant biomarker for the prognosis of HCC patients.

## Material and Methods

### Study Design and patients

One hundred and twenty two patients with HCC who were treated in Tohoku University Hospital were enrolled in this study. They had liver disease due to viral hepatitis such as HBV and/or HCV infection, alcoholic hepatitis, non-alcoholic steatohepatitis, primary biliary cirrhosis or autoimmune hepatitis. Peripheral blood samples were obtained in heparin-containing tubes before treatment with surgery, TACE, RFA, radiation therapy, hepatic arterial infusion chemotherapy (HAIC) or any systemic chemotherapy. Permission for the study was obtained from the Ethics Committee at Tohoku University Graduate School of Medicine following ethical guidelines of the 1975 Declaration of Helsinki. Written informed consent was obtained from all patients enrolled in this study. These procedures were carried out in accordance with the approved guidelines, and all experimental protocols were approved by the Ethics Committee at Tohoku University Graduate School of Medicine.

### Isolation of PBMCs and tumor infiltrating lymphocytes

PBMCs were isolated from fresh heparinized blood by Lymphoprep (AXIS-SHIELD, Oslo, Norway) density gradient centrifugation. Peripheral blood of HCC patients was collected prior to treatment initiation. Fifty-three healthy volunteers and fifty-two patients with chronic hepatitis including twenty with HBV and thirty-two with HCV infection served as controls. TILs and liver-infiltrating lymphocytes (LILs) were mechanistically isolated from resected liver cancer tissue and normal liver tissue. PBMCs, TILs and LILs were stocked with cell banker (Takara Bio, Shiga, Japan) at −80 °C.

### Flow cytometry analysis

To determine the frequency of PDL1^+^MDSCs, multicolor fluorescene-activated cell sorting (FACS) analysis was done using the following antibodies: anti-human CD11b, CD14, CD33, HLA-DR and PD-L1. All monoclonal antibodies used in this study were purchased (BioLegend, San Diego, CA). Cells were incubated at 4 °C for 15 min. After incubation, each sample was washed twice with phosphate-buffered saline (PBS). Pellets were re-suspended in 500 μl of 2% paraformaldehyde-PBS. Isotype-matched antibodies were used with all the samples as controls. Flow cytometry was performed on BD LSRFortessa (BD Bioscience, Franklin Lakes, New Jersey). The data were analyzed using FlowJo software (Tree Star, Ashland, Ohio).

### Sorting of Immature myeloid cells

Fresh PBMCs were isolated from healthy individuals. To isolate immature myeloid cells (ImMCs), PBMCs were stained with antihuman CD33, HLA-DR following the method described above. ImMCs were sorted into CD33^dim^HLA-DR^−^ cells using BD FACS Aria II cell sorting system (BD, Franklin Lakes, New Jersey). Sorted ImMCs were stored in RPMI (Life Technologies, San Diego, CA) and immediately used in the co-incubation study by the following method.

### Cell culture

Hep3B, Li7, PLC, HepG2 and Huh7 were obtained from the Cell Resource Center for Biomedical Research, Tohoku University at 2008. AGS and HT29 were purchased from ATCC at 2008. 786-O was purchased from ATCC at 2013. A trans-membrane of 0.4 μm pore size (Corning Life Science, Pittsburgh, PA) was used for the analysis of the soluble factors secreted by liver cancer cell lines. The upper chamber included PBMCs (1 × 10^6^ cells/ml) from healthy donors, and the lower chamber included several different liver cancer cell lines (1 × 10^5^ cells/ml) such as Hep3B, Li7, PLC, HepG2 and Huh7. After 72 hours coincubation at 37 °C under an atmosphere of 5% CO_2_, multicolor FACS analysis was performed as described above.

### Induction of MDSCs by using recombinant human M-CSF or VEGF

After the isolation of PBMCs from a healthy subject, PBMCs were stimulated with recombinant human M-CSF (R&D, Minneapolis, MN) 1 ng/mL or recombinant human VEGF (R&D, Minneapolis, MN) 2 ng/mL for 72 hours. The stimulated PBMCs were stained with the various antibodies described above and analyzed by FACS.

### RNA isolation and Real-Time PCR reaction

Total cellular RNA was extracted from the liver cancer cell lines described above using an RNeasy Mini Kit (Qiagen, Valencia, CA) according to the manufacturer’s instructions. Contaminating small DNA was removed by DNase I digestion using an RNase-free DNase system (Qiagen). The amount of extracted RNA was measured by NanoDrop ND-1000 (NanoDrop Technologies, Rockland, DE). The commercially available primers and probe for the amplification of CSF-1, CSF-2, CSF-3, IL-1, IL-6, CCL2, VEGFA, S100A8 and S100A9 and GAPDH were purchased from Perkin-Elmer Applied Biosystems. The data are shown in 2^−ΔΔCt^ format.

### ELISA assay

Plasma was isolated during PBMC separation and supernatants were collected from confluent cell line cultures. They were maintained in −80 °C. The samples were resolved at the same time, and the concentrations of M-CSF and VEGF in these samples were measured using ELISA assay kit (R&D, Minneapolis, MN) following the manufacturer’s instructions.

### Statistics

Data are expressed as mean value ± standard error of the mean for percentages. Statistical analysis was done using Student’s *t-test* and one-way ANOVA to assess the differences between the study groups. Statistical significance was defined as *p* < 0.05.

## Results

### PD-L1^+^MDSCs are increased in the peripheral blood from HCC patients

**Figure 1:**
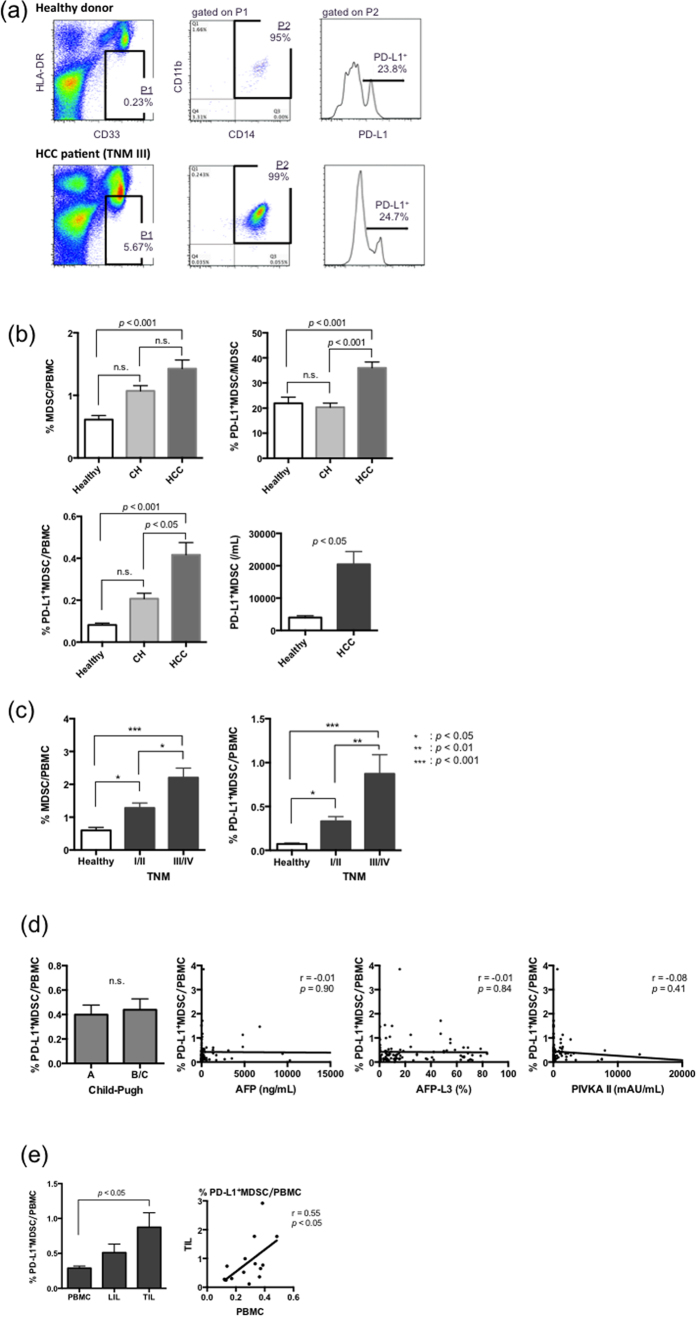
PD-L1^+^MDSCs are increased in the peripheral blood from HCC patients. (**a)** Representative dot plots of a normal healthy donor (upper panel) and a HCC patient with TNM III (lower panel) are shown. The dot plots show CD33^+^HLA-DR^low/−^CD11b^+^CD14^+^ MDSCs. (**b**) There were 53 healthy donors, 52 chronic hepatitis patients (CH) and 122 HCC patients. (upper left panel) The percentages of MDSCs in HCC patients were significantly higher than those in healthy donors (*p* < 0.001), and there were no significant differences in the percentages of MDSCs in the CH patients comparison with the healthy donors and HCC patients. (upper right panel) PD-L1^+^ cells in total MDSCs in HCC patients were significantly higher than those in heatlhy subjects and CH patients (*p* < 0.001). (lower left panel) The percentages of PD-L1^+^MDSCs in HCC patients were significantly higher than those in healthy donors (*p* < 0.001) and patients with chronic hepatitis (*p* < 0.05). (lower right panel) Circulating PD-L1^+^MDSCs were at significantly higher levels in HCC patients than in healthy donors (*p* < 0.05). (**c**) The percentages of MDSCs and PD-L1^+^MDSCs significantly increased as the stage of HCC advanced. (**d**) The Child-Pugh grade and the level of cancer biomarkers of the HCC patients were not related to the percentages of PD-L1^+^MDSCs. (**e**) (upper panel) The percentages of PD-L1^+^MDSCs were significantly higher in TILs than in PBMCs (*p* < 0.05). (lower panel) The percentages of PD-L1^+^MDSCs in TILs and PBMCs showed a positive correlation (*p* < 0.05).

We analyzed the presence of PD-L1^+^MDSCs in the peripheral blood from HCC patients with various stages. The clinical characteristics of all HCC patients are shown (Table 1). The characteristics of the HCC patients at post-treatment and recurrence are also shown (Table 2). The percentages of PD-L1^+^MDSCs were calculated as the percentage of total PBMCs. Representative dot plots of a normal healthy donor and an HCC patient with TNM III are shown in Fig. 1a. The percentages of MDSCs are significantly higher in the HCC patients compared with healthy donors (mean 1.42% vs 0.59%; *p* < 0.001) (Fig. 1b). PD-L1 positive cells in total MDSCs are significantly higher in HCC patients compared to healthy donors (mean 36.0% vs 23.8%; *p* < 0.001) (Fig. 1b). PBMCs from HCC patients had significantly higher percentages of PD-L1^+^MDSCs compared to those from healthy subjects (mean 0.41% vs 0.079%; *p* < 0.001) and patients with chronic hepatitis (mean 0.41% vs 0.20%; *p* < 0.05) (Fig. 1b). Similarly, a significantly higher level of circulating PD-L1^+^MDSCs was observed in HCC patients compared to normal volunteers (mean 20434/mL vs 4004/mL; *p* < 0.05) (Fig. 1b). Then, we divided HCC patients by the clinical cancer stage. The percentages of MDSCs significantly increased as the stage of HCC advanced (healthy donor vs TNM I/II vs TNM III/IV; 0.59% vs 1.28% vs 2.20%) (healthy donor vs TNM I/II; *p* < 0.05, healthy donor vs TNM III/IV; *p* < 0.001, TNM I/II vs TNM III/IV; *p* < 0.05) (Fig. 1c). Similarly, the percentages of PD-L1^+^MDSCs significantly increased as the stage of HCC advanced (healthy donor vs TNM I/II vs TNM III/IV; 0.079% vs 0.33% vs 0.87%) (healthy donor vs TNM I/II; *p* < 0.05, healthy donor vs TNM III/IV; *p* < 0.001, TNM I/II vs TNM III/IV; *p* < 0.01) (Fig. 1c). In addition, we also analyzed the correlation between the percentages of PD-L1^+^MDSCs and Child-Pugh grade and serum concentration of well-known hepatocellular cancer biomarkers (AFP, AFP-L3 and PIVKA II). The difference in the Child-Pugh grade was unrelated to the percentages of PD-L1^+^MDSCs (Child-Pugh A vs B/C; 0.39% vs 0.43%; *p* = 0.738) (Fig. 1d). Similarly, the level of cancer biomarkers did not correlate with the percentages of PD-L1^+^MDSCs (Fig. 1d).

### TILs contained remarkably high percentages of PD-L1^+^MDSCs

We examined the frequency of PD-L1^+^MDSCs in TILs and LILs using resected liver cancer tissue. TILs and LILs were prepared from the fresh, surgically excised liver cancer tissue of 14 HCC patients with various stages. The representative dot plots of TILs and PBMCs are shown in Supplement Figure 1. The percentages of PD-L1^+^MDSCs were high in terms of TILs, LILs and PBMCs (PBMCs vs LILs vs TILs; 0.28% vs 0.51% vs 0.87%), and the percentages of PD-L1^+^MDSCs in TILs were significantly higher than those in PBMCs (*p* < 0.05) (Fig. 1e). LILs contained mildly increased PD-L1^+^MDSCs, but the percentages of PD-L1^+^MDSCs in LILs did not reach statistical significance (PBMCs vs LILs; *p* = 0.50, LILs vs TILs; *p* = 0.17) (Fig. 1e). The percentages of PD-L1^+^MDSCs in TILs and PBMCs showed a positive correlation (r = 0.55, *p* < 0.05) (Fig. 1e).

### Dynamics of PD-L1^+^MDSCs before and after curative treatment

**Figure 2:**
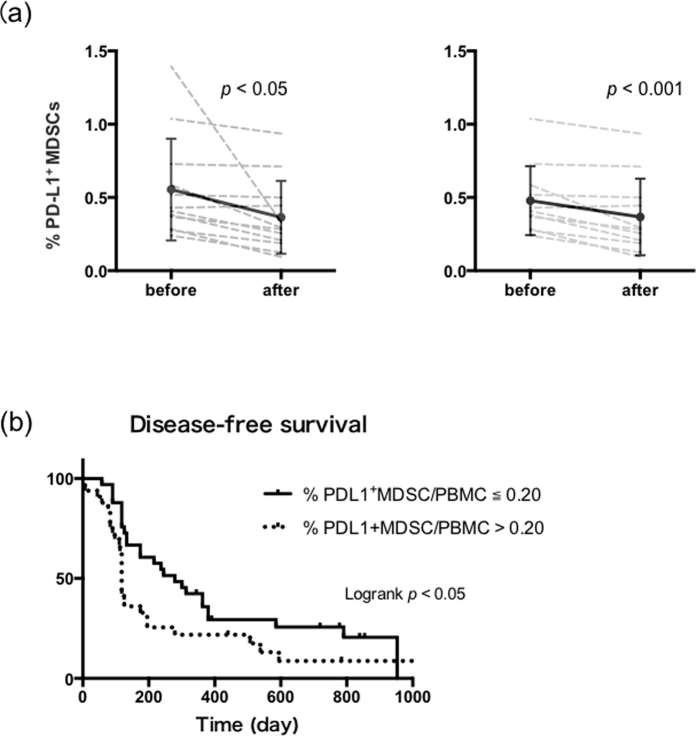
The levels of PD-L1^+^MDSCs were associated with a prognosis. (**a**) The percentages of PD-L1^+^MDSCs were significantly reduced by curative treatment for HCC (n = 12, *p* < 0.05) (left panel). The percentages of PD-L1^+^MDSCs were also significantly reduced by curative treatment for HCC after the outlier with an extreme reduction of MDSCs frequency is omitted. (n = 11, *p* < 0.001) (right panel). (**b**) Patients with high levels of PD-L1^+^MDSCs had significantly shorter disease-free survival periods than those with low level (n55, *p* < 0.05).

We examined the percentages of PD-L1^+^MDSCs before and after curative treatment for HCC, including RFA and TACE, in twelve patients. Peripheral blood samples were collected just before treatment initiation and 2 weeks after treatment. The percentages of PD-L1^+^MDSCs were significantly reduced by curative treatment for HCC (before vs after; 0.54% vs 0.40%; *p* < 0.05) (Fig. 2a). Then, we divided 55 patients with HCC, who received curative treatments, into two groups according to the percentages of PD-L1^+^MDSCs. The low PD-L1^+^MDSCs group included patients who had percentages of PD-L1^+^MDSCs ≦ 0.20%, and the high PD-L1^+^MDSCs group included those who had percentages of PD-L1^+^MDSCs > 0.20%. This threshold was the intermediate frequency of PD-L1^+^MDSCs before the treatment of HCC. The low PD-L1^+^MDSCs group had significantly longer disease-free survival than the high PD-L1^+^MDSCs group (median 279 days vs 118 days; *p* < 0.05) (Fig. 2b). Moreover, we divided the HCC patients of TNM stage I who received curative treatment into two groups by the same methods, and similar results were obtained (Supplement Figure 2).

### The differentiation of PD-L1^+^MDSCs was induced by soluble factors from HCC

**Figure 3:**
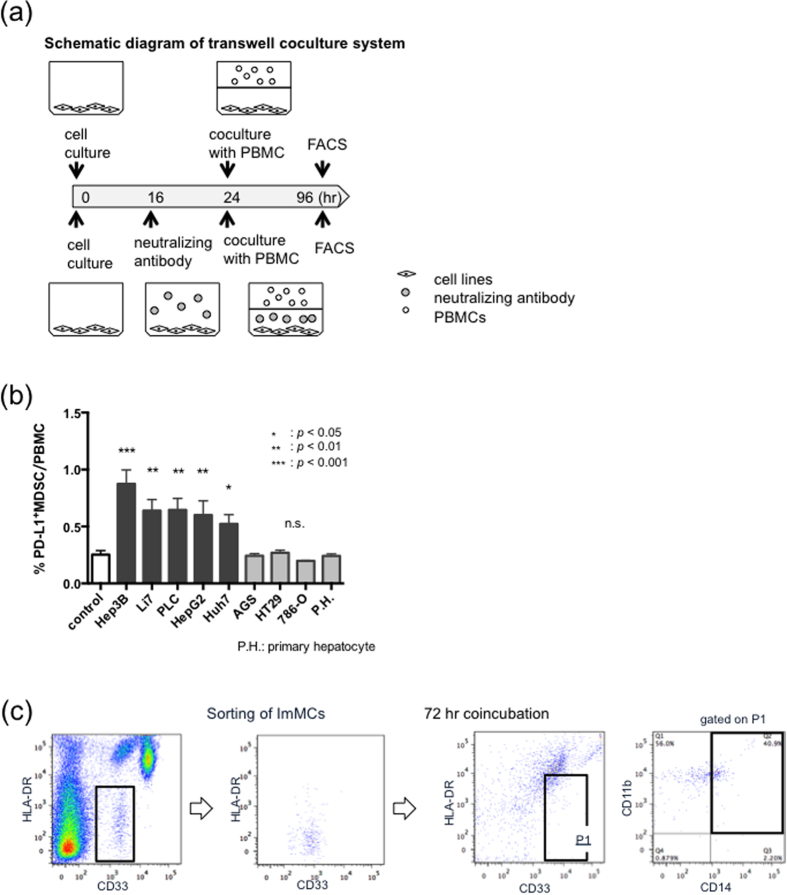
Soluble factors from HCC played an important role in the differentiation of PD-L1^+^MDSCs. (**a**) This is a schematic diagram of the transwell coculture system. Upper image shows a normal coincubation and lower image shows a coincubation using a neutralizing antibody. (**b**) The percentages of PD-L1^+^MDSCs were significantly increased after 72 hours coincubation with several cancer cell lines compared with control, primary hepatocyte and other cancer cell lines. (**c**) A part of the ImMCs showed a phenotype similar to MDSCs after coincubation with ImMCs and cancer cell lines. (**d**) The percentages of PD-L1^+^MDSCs under the stimulation of recombinant human M-CSF or VEGF were significantly higher than in those without cytokine stimulation.

After 72 hours co-incubation with several liver cancer cell lines (Fig. 3a), the percentages of PD-L1^+^MDSCs were significantly increased compared with control (control 0.25%, Hep3B 0.87%; *p* < 0.001, Li7 0.63%; *p* < 0.01, PLC 0.64%; *p* < 0.01, HepG2 0.60%; *p* < 0.01, Huh7 0.52%; *p* < 0.05) (Fig. 3b). After co-incubation with primary hepatocytes or other cancer cell lines such as AGS, HT29 and 786-O, the percentages of PD-L1^+^MDSCs did not differ compared with control (AGS 0.24%; *p* = 0.95, HT29 0.26%; *p* = 0.94, 786-O 0.19%; *p* = 0.82) (Fig. 3b). It was reported that MDSCs could be differentiated from ImMCs under the influence of various factors. To confirm that some kinds of soluble factors induced the differentiation of MDSCs from ImMCs, we co-incubated ImMCs and Hep3B, which induced PD-L1^+^MDSCs most strongly in the above-described co-cultivation experiment. After 72 hours co-incubation, a part of the ImMCs showed a phenotype similar to MDSCs (Fig. 3c).

### M-CSF and VEGF were important factors that promoted the induction of PD-L1^+^MDSCs

**Figure 4:**
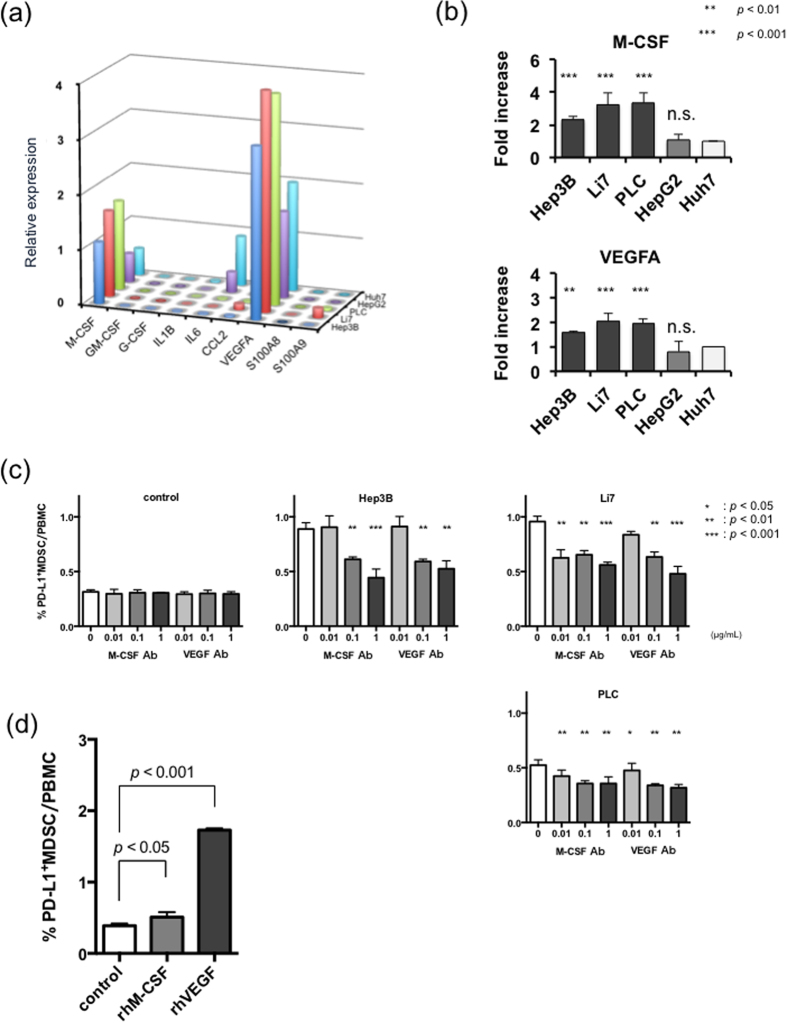
M-CSF and VEGF were key factors that promoted the induction to PD-L1^+^MDSCs. (**a**) The results of the real-time PCR reaction. (**b**) The expression of M-CSF and VEGFA was significantly higher in the cell lines that strongly induced PD-L1^+^MDSCs. (**c**) The percentages of PD-L1^+^MDSCs were reduced after coincubation using neutralizing antibody of M-CSF and VEGF. (**d**) The induction of MDSCs by using recombinant human M-CSF or VEGF.

We extracted total RNA from various liver cancer cell lines. Then, we carried out real-time PCR to quantify various mRNAs reported as factors that could induce MDSCs. We tried to detect factors that could induce the differentiation of PD-L1^+^MDSCs in the co-incubation experiment. The results of the real-time PCR experiments are shown in Fig. 4a. The expression patterns of candidate factors differed among the liver cancer cell lines. However, the expression levels of M-CSF and VEGFA were significantly higher in the cell lines that could strongly induce PD-L1^+^MDSCs such as Hep3B, Li7 and PLC compared with Huh7, which showed the lowest PD-L1^+^MDSCs induction (*p* < 0.01) (Fig. 4b). Then, we co-incubated PBMCs and liver cancer cell lines using neutralizing antibody of M-CSF and VEGF to confirm that the induction of PD-L1^+^MDSCs could be promoted by M-CSF and VEGF. As the concentration of neutralizing antibody increased, the PD-L1^+^MDSCs ratio was dose-dependently decreased (Fig. 4c and Supplement Figure 3). Then, we tried to analyze whether the recombinant human M-CSF or VEGF could induce PD-L1^+^MDSCs. The frequencies of PD-L1^+^MDSCs under the stimulation of recombinant human M-CSF or VEGF were significantly higher than those without cytokine stimulation (control 0.38%, rh-M-CSF 0.50% (p < 0.05) and rh-VEGF 1.72% (p < 0.001)) (Fig. 4d). Also, the supernatant of the co-incubation experiments was collected for the M-CSF and VEGF array. With Hep3B, Li7 and PLC, which strongly induced PD-L1^+^MDSCs, the supernatant M-CSF and VEGF concentration was higher than that with the other liver cancer cell lines (Supplement Figure 4a and b). By using neutralizing antibody of M-CSF and VEGF, the supernatant M-CSF and VEGF concentration decreased dose-dependently (Supplement Figure 4a and b). These results demonstrated that M-CSF and VEGF were associated with the induction of PD-L1^+^MDSCs.

We also measured the M-CSF and VEGF density in the peripheral blood of HCC patients in order to confirm where M-CSF and VEGF induced PD-L1^+^MDSCs. The serum M-CSF and VEGF concentration was not associated with the Child-Pugh grade and tumor stage (Supplement Figure 5a and b). Also, The serum M-CSF and VEGF density did not correlate with the percentages of PD-L1^+^MDSCs and was unrelated to the disease-free survival (Supplement Figure 5a and b). These results showed that PD-L1^+^MDSCs were mainly induced in the cancer microenvironment, not but in the peripheral blood.

## Discussion

MDSCs are expanded in pathological conditions such as malignancy or infection, and suppress antitumor immunity^26^,^27^. PD-L1 expression on activated monocytes and macrophages was associated with the disease progression and poor survival of patients^28^. However, little is known about the function of PD-L1^+^MDSCs and the relationship between PD-L1^+^MDSCs and the prognosis of HCC.

In this study, we showed that PD-L1^+^MDSCs were induced by MCSF, VEGF released by a liver cancer cell line. It has been reported that MDSCs in peripheral blood are increased in patients with hepatocellular carcinoma^29^,^30^, and we have similar results in this study. Our data demonstrated that the frequency of PD-L1^+^MDSCs increased in HCC patients. Moreover, the frequency of PD-L1^+^MDSCs increased with the progression of HCC. In addition to this study, other groups reported that there were no significant differences in the frequency of PD-L1^+^MDSCs between healthy donors and chronic hepatitis patients. They demonstrated that the increase in MDSCs was only correlated with tumor progression, but not with hepatic fibrosis or the disease activity of chronic liver disease^30^. On the other hand, other research groups reported that M-CSF was associated with hepatic fibrosis in chronic hepatitis C and non-alcoholic fatty liver disease patients^31^,^32^. Moreover, they found that M-CSF could be produced from hepatocytes with persistent HCV infection^31^. In this study, there was a significant difference in the percentages of PD-L1^+^MDSCs between patients with HCC and those with chronic hepatitis. This result showed that soluble factors from HCC might be important for the induction of MDSCs. On the other hand, the percentages of PD-L1^+^MDSCs in chronic hepatitis patients were higher than those in healthy donors, although not significantly. In addition, there were significantly higher percentages of PD-L1^+^MDSCs in HCC patients with curative treatment than in chronic hepatitis patients. HCC patients have more severe liver fibrosis compared to chronic hepatitis patients. Therefore, liver fibrosis might influence the induction of PD-L1^+^MDSCs.

In evaluating accurately the cancer immunity mechanism, particularly the influence on the metastatic lesions, it is important to identify whether the induction of PD-L1^+^MDSCs is caused in the tumor microenvironment or systemically. We hypothesized two possibilities: one was that PD-L1^+^MDSCs that increased in the tumor microenvironment migrated to the peripheral blood, and the other was that PD-L1^+^MDSCs were generated in the peripheral blood of patients with HCCs. Therefore, co-cultivation experiments were carried out to determine the unidentified factors that could induce PD-L1^+^MDSCs. The frequency of PD-L1^+^MDSCs was increased after co-incubation with the liver cancer cell lines, particularly Hep3B, Li7 and PLCs. We extracted total RNA from the liver cancer cell lines, and real-time PCR was carried out to quantify various mRNAs. The expression levels of M-CSF and VEGFA were high in the cell lines that could strongly induce PD-L1^+^MDSCs, so we assumed that M-CSF and VEGFA secreted from these cell lines played an important role in the induction of PD-L1^+^MDSCs. To confirm this hypothesis, we performed a co-incubation experiment using neutralizing antibody of M-CSF and VEGF. After co-incubation, the induction of PD-L1^+^MDSCs could be dose-dependently inhibited by VEGF and M-CSF neutralizing antibodies. These results demonstrated that M-CSF and VEGF played a pivotal role in the induction of PD-L1^+^MDSCs. However, the serum amount of M-CSF and VEGF in HCC patients did not correlate with the percentages of PD-L1^+^MDSCs, the hepatic reserve, tumor progression and disease-free survival. Therefore, it was thought that the hypothesis that PD-L1^+^MDSCs were generated in the peripheral blood was incorrect. However, the percentages of PD-L1^+^MDSCs in TILs were higher that those in PBMCs, and the percentages of PD-L1^+^MDSCs in TILs and PBMCs showed a positive correlation. Resent studies showed that cytokines released by tumors are induced by MDSCs^33^,^34^. Our data suggested that PD-L1^+^MDSCs were induced in the tumor microenvironment, but little is known about correlations between the quantity of cytokines in the tumor local site and those in the peripheral blood. Therefore, further study of cytokines in the tumor microenvironment is necessary.

There are studies of antibody therapy and antitumor vaccine therapy that targeted immune checkpoint molecules in various cancers^35^,^36^,^37^. Anti PD-1 antibody treatment or anti PD-L1 antibody treatment for lung cancer or malignant melanoma are used in clinical practice^38^,^39^,^40^. It was reported that MDSCs in the tumor tissue express PD-L1 in a mouse liver cancer model, and suppression of T cell function through MDSCs was removed by blocking PD-L1^41^,^42^. Similar to these preliminary studies, we found that PD-L1^+^MDSCs were related to hepatocellular carcinoma extension in HCC patients for the first time. Our data showed that T cells from HCC patients were exhausted compared with healthy donors, and this result demonstrated that this exhaustion was caused by contact with PD-L1^+^MDSCs or soluble factors from PD-L1^+^MDSCs. Therefore, in human hepatocellular carcinoma, PD-L1^+^MDSCs play important roles in the suppression of T cell function and tumor growth, and it is suggested that anti PD-L1 antibody treatment could be effective for hepatocellular carcinoma.

AFP, AFP-L3 and PIVKA II are widely used and broadly known biomarkers for HCC management^24^. In this study, PD-L1^+^MDSCs were not related to these biomarkers. That is why tumor cells producing M-CSF and VEGF are different from tumor cells producing AFP. This suggests that PD-L1^+^MDSCs reflect the quantity of other characteristics of cancer cells. The percentages of PD-L1^+^MDSCs were significantly reduced by curative treatment for HCC, and the frequency of PD-L1^+^MDSCs affected the disease-free survival. These data suggest that more sensitive screening of HCC may be enabled by monitoring PD-L1^+^MDSCs together with conventional tumor markers. Further research of the long-term dynamics of PD-L1^+^MDSCs is necessary. The induction of PD-L1^+^MDSCs by differences in the etiology was not investigated sufficiently in this study, because there were many cases of HCC patients who were infected with HCV. A prospective study by multicenter with larger numbers of patients is needed. A recent study showed that IL18 plays an important role in the accumulation of MDSCs in the liver^43^, and IL18 expression was increased in an HBV-replicating hepatoma cell line in our preliminary experiment^10^. Further study of the factors increasing PD-L1^+^MDSCs is necessary.

## Additional Information

**How to cite this article:** Iwata, T. *et al*. PD-L1^+^MDSCs are increased in HCC patients and induced by soluble factor in the tumor microenvironment. *Sci. Rep.* **6**, 39296; doi: 10.1038/srep39296 (2016).

**Publisher’s note:** Springer Nature remains neutral with regard to jurisdictional claims in published maps and institutional affiliations.

## Supplementary Material

Supplementary Figure

## Figures and Tables

**Table 1:** Characteristics of HCC patients.

Characteristics of patients	Number/Value
**Total number**	122
**Age (years)**	63.8 ± 9.4 (mean ± SD)
**Gender (Male:Female)**	83:39
**Etiology**	
B	19
C	75
B+C	9
NBNC	19
alcohol	11
NASH	2
PBC	1
unknown	5
**Child-Pugh Class**	
A	73
B	47
C	2
**TNM UICC7**	
I	48
II	55
IIIA	4
IIIB/IIIC/IVA	0
IVB	15
**Treatment**	
OP	14
RFA	9
TACE	77
RT	10
TACE+RFA	5
HAIC	2
sorafenib	3
BSC	2
**Laboratory Data**	
T-BIL (mg/dL)	1.15 (0.3–3.5)
AST (IU/L)	46 (15–145)
ALT (IU/L)	37 (13–135)
ALB (g/dL)	3.3 (2.0–4.3)
PT (%)	82.2 (53.4–120)
WBC (/L)	3900 (1600–13500)
Hb (g/dL)	13 (9.3–16.2)
PLT (10^3^/L)	100 (39–318)
AFP (ng/mL)	39.8 (3.1–17166)
AFP-L3 (%)	11 (0.5–83.5)
PIVKA II (mAU/mL)	45 (9–20300)

Hepatitis B virus (B), heatitis C virus (C), operation (OP), radiofrequency ablation (RFA), trans arterial chemo-embolization (TACE), radiation therapy (RT), hepatic artery infusion chemotherapy (HAIC), best supportive care (BSC), total bilirubin (T-BIL), aspirate aminotransferase (AST), alanine aminotransferase (ALT), albumin (ALB), prothrombin time (PT), white blood cell (WBC), hemoglobin (Hb), platelet (PLT), alpha-fetoprotein (AFP), prothrombin induced by vitamin K absence II (PIVKA- II).

**Table 2:** Characteristics of HCC patients at the post-treatment and recurrence.

Characteristics of patients	Value
**Laboratory Data (post-treatment)**	
T-BIL (mg/dL)	1.04 (0.4–2.5)
AST (IU/L)	40.7 (21–96)
ALT (IU/L)	32.7 (5–118)
ALB (g/dL)	3.47 (2.7–4.3)
PT (%)	102.3 (61–120)
WBC (/L)	4150 (2299–8500)
Hb (g/dL)	11.1 (8.8–13.6)
PLT (10^3^/L)	144 (36–387)
AFP (ng/mL)	109.4 (2.3–568)
AFP-L3 (%)	14.5 (0.5–78.9)
PIVKA II (mAU/mL)	206.8 (10–1920)
**Laboratory Data (recurrence)**	
T-BIL (mg/dL)	1.36 (0.4–2.9)
AST (IU/L)	60.2 (18–145)
ALT (IU/L)	47.9 (13–135)
ALB (g/dL)	3.25 (2–4.2)
PT (%)	79.6 (60–120)
WBC (/L)	3763 (1600–6400)
Hb (g/dL)	13.0 (9.8–16.1)
PLT (10^3^/L)	102.9 (39–318)
AFP (ng/mL)	1003.0 (6.1–15099)
AFP-L3 (%)	30.2 (0.5–78.5)
PIVKA II (mAU/mL)	643.5 (13–7530)
