# Supplementary material for: PD-L1+MDSCs are increased in HCC patients and induced by soluble factor in the tumor microenvironment
Source: Sci Rep. 2016 Dec 14;6:39296. doi: 10.1038/srep39296 (PMC5155242; doi:10.1038/srep39296)
Supplement: Supplementary Figure [file srep39296-s1.pdf]

PD-L1$^+$MDSCs are increased in HCC patients and induced by soluble factor in the tumor microenvironment

Tomoaki Iwata[1#], Yasuteru Kondo[1, 2, #*], Osamu Kimura[1], Tatsuki Morosawa[1], Yasuyuki Fujisaka[1], Teruyuki Umetsu[1], Takayuki Kogure[1], Jun Inoue[1], Yu Nakagome[1], Tooru Shimosegawa[1]

1. Division of Gastroenterology, Tohoku University Hospital, 1-1 Seiryo, Aoba, Sendai City, Miyagi, Japan 980-8574
2. Department of Hepatology, Sendai Kousei Hospital, 4-15 Hirose, Aoba, Sendai City, Miyagi, Japan 980-0873

[#] The authors are equally contributed to this manuscript.

* Corresponding Author
Yasuteru Kondo, MD/PhD
Chief Director
Department of Hepatology
Sendai Kousei Hospital
E-mail:yasuteru@ebony.plala.or.jp

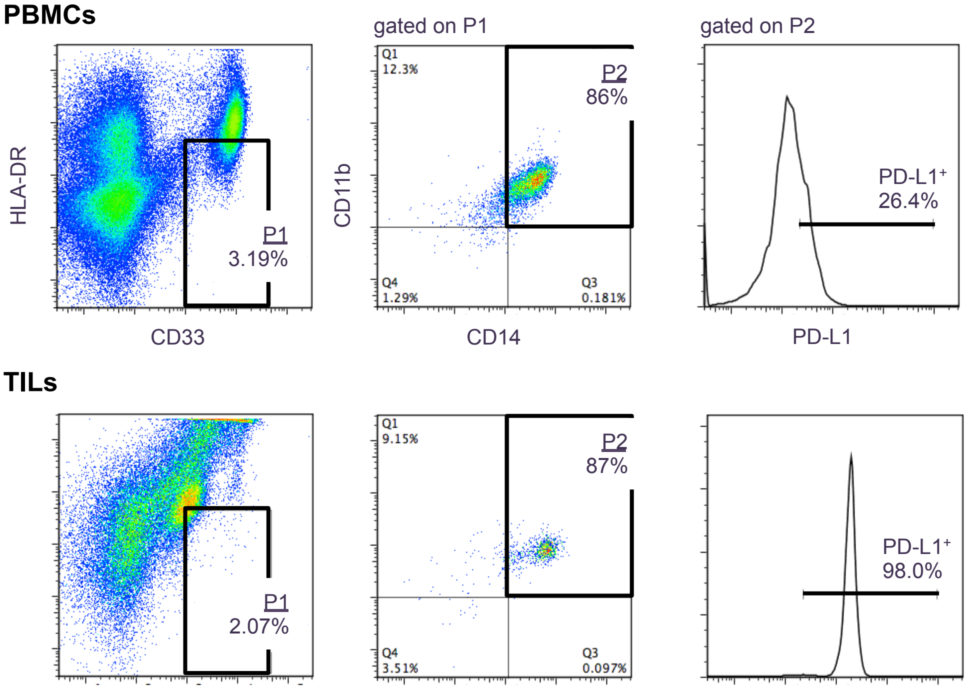

Supplement Figure 1. Representative dot plots of PBMCs and TILs from the HCC patients treated by operation.

Supplement Figure 1

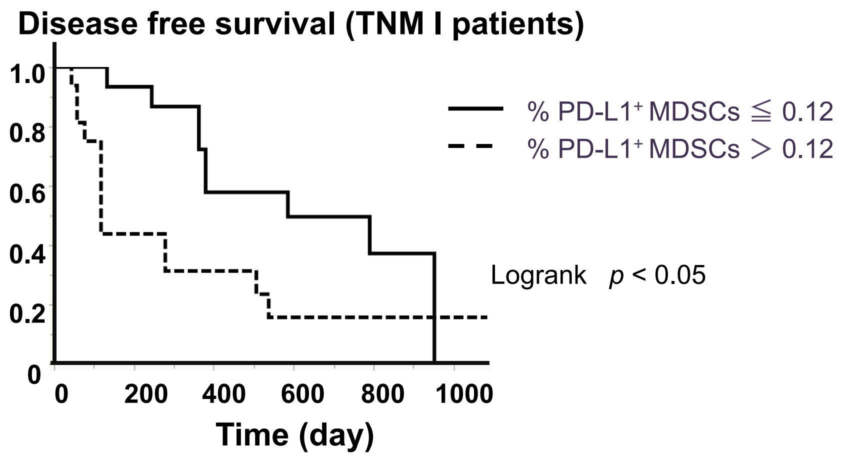

Supplement Figure 2. Patients with high levels of PD-L1^{+}MDSCs at the pre-treatment had significantly shorter disease-free survival periods than those with low levels at the pre-treatment.

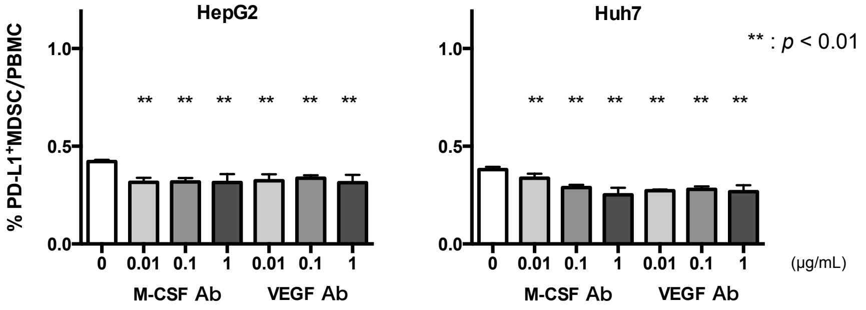

Supplement Figure 3. The percentages of PD-L1^{+}MDSCs were reduced after coincubation using neutralizing antibody of M-CSF and VEGF.

Supplement Figure 3

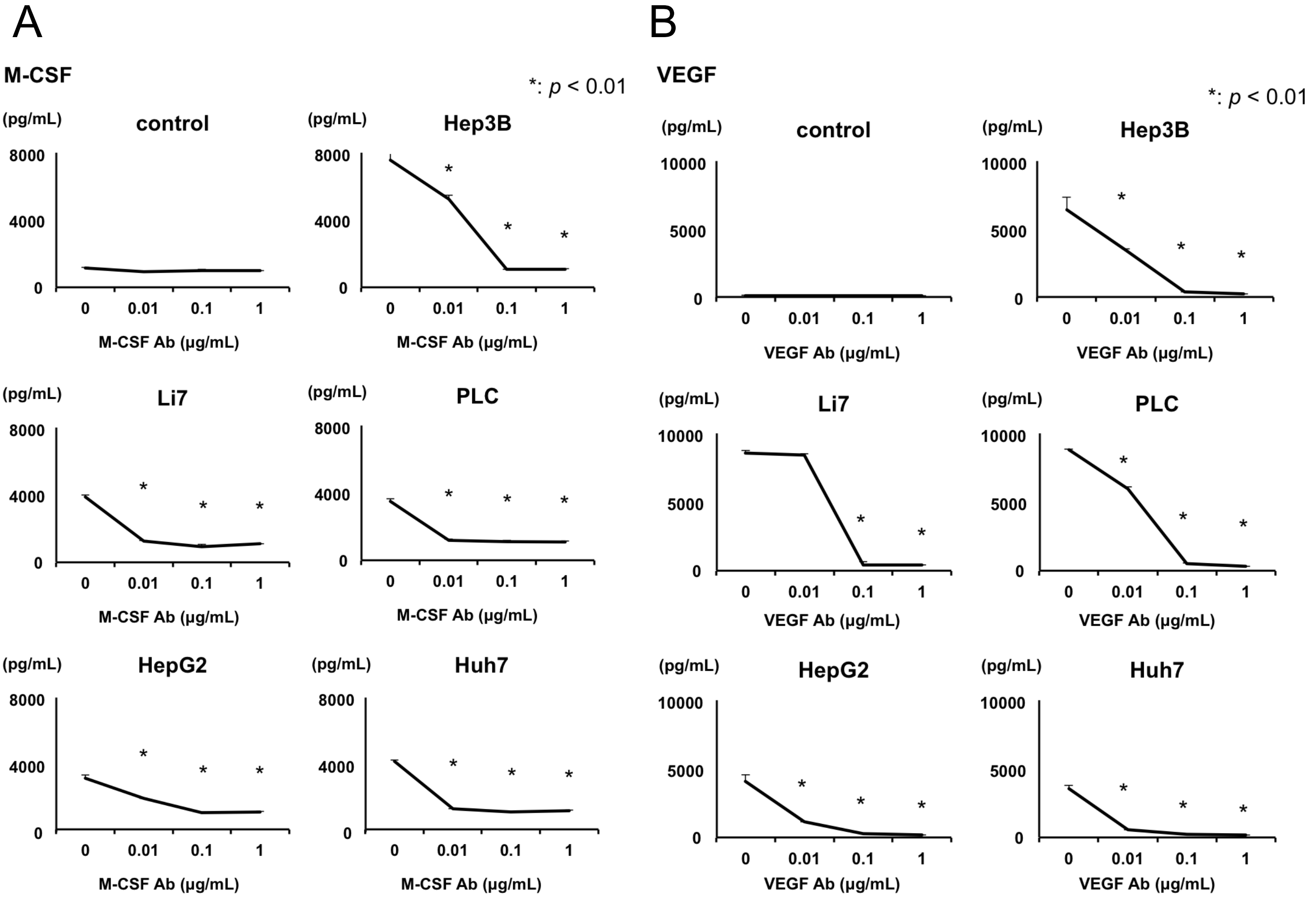

Supplement Figure 4. Supernatant M-CSF and VEGF concentration decreased by using neutralizing antibody.
A. This shows the change in the supernatant M-CSF concentration. **B.** This shows the change in the supernatant VEGF concentration.

Supplement Figure 4

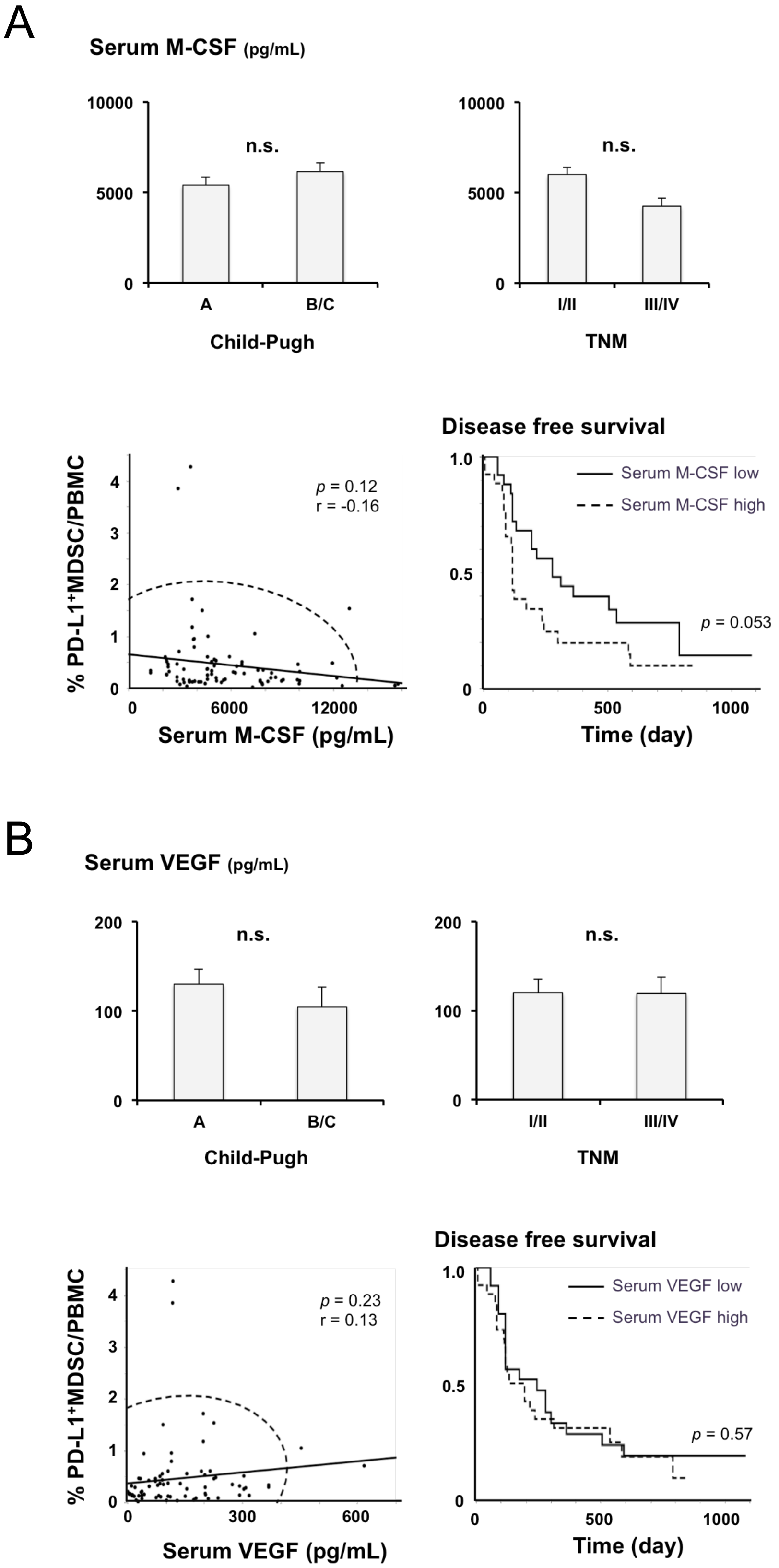

Supplement Figure 5. The serum M-CSF and VEGF concentration was not associated with the percentages of PD-$L1^+$MDSCs.
A. The serum M-CSF concentration was not associated with the clinical characteristics of HCC patients or the prognosis. **B.** The serum VEGF concentration showed similar results.

Supplement Figure 5
